# Supplementary material for: Multi-modal generative modeling for joint analysis of single-cell T cell receptor and gene expression data
Source: Nat Commun. 2024 Jul 3;15:5577. doi: 10.1038/s41467-024-49806-9 (PMC11220149; doi:10.1038/s41467-024-49806-9)
Supplement: Supplementary file 1 — Supplementary Information [file 41467_2024_49806_MOESM1_ESM.pdf]

Multi-modal generative modeling for joint analysis of single-cell T-cell receptor and gene expression data

Felix Drost[1,2], Yang An[1,3], Irene Bonafonte-Pardàs[1], Lisa M Dratva[4], Rik GH Lindeboom[5], Muzlifah Haniffa[4,6], Sarah A Teichmann[4,7], Fabian Theis[1,2,3], Mohammad Lotfollahi[1,4,†,⋆], Benjamin Schubert[1,3,†,⋆]

1 Computational Health Center, Helmholtz Munich, Ingolstädter Landstraße 1, 85764 Neuherberg, Germany
2 School of Life Sciences Weihenstephan, Technical University of Munich, Alte Akademie 8, 85354 Freising, Germany
3 School of Computation, Information and Technology, Technical University of Munich, Boltzmannstraße 3, 85748 Garching bei München, Germany
4 Wellcome Sanger Institute, Wellcome Genome Campus, Hinxton, Cambridge, UK
5 The Netherlands Cancer Institute, Plesmanlaan 121, 1066 CX Amsterdam, The Netherlands
6 Biosciences Institute, Newcastle University, Newcastle upon Tyne NE2 4HH, UK
7 Department of Physics, Cavendish Laboratory, University of Cambridge, 19 JJ Thomson Avenue, Cambridge, UK

† Correspondence to:

ml19@sanger.ac.uk
benjamin.schubert@helmholtz-muenchen.de

⋆ These authors jointly supervised this work.

Supplementary Figures

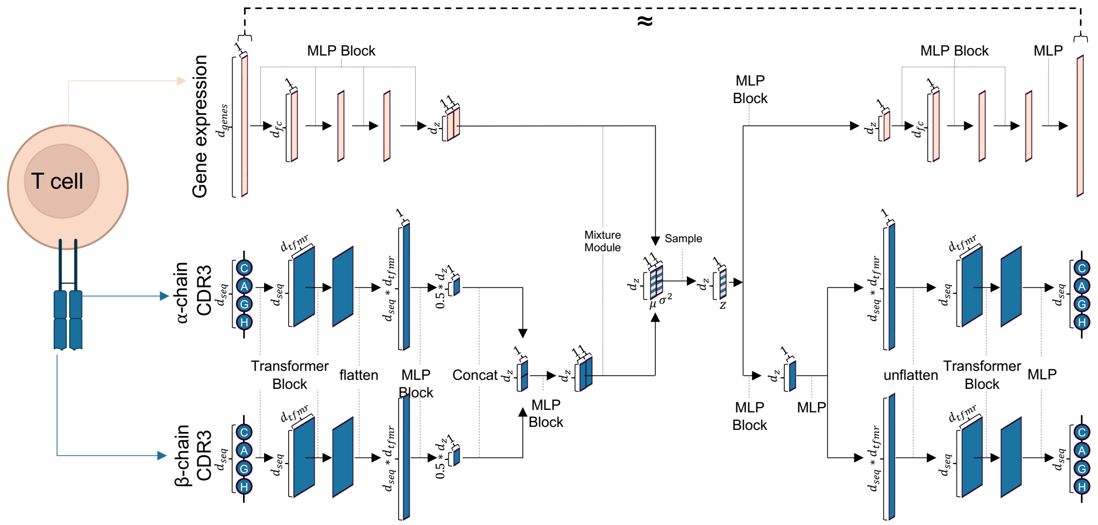

Supplementary Figure 1 | Detailed overview of the mvTCR architecture. The Variational Autoencoder (VAE) model receives a gene expression vector, and the tokenized CDR3α- and CDR3β-amino acid sequence. The gene expression is transformed via a stack of fully connected blocks, while the TCR sequences are encoded using two separate transformer blocks, followed by a fully connected block. The modalities are fused by one of three mixture modules (Mixture-of-Export, Product-of-Expert, or Concatenation). After variational inference via the reparemetrization trick, the original input is reconstructed by encoding networks following the reverse architecture of the encoding networks.

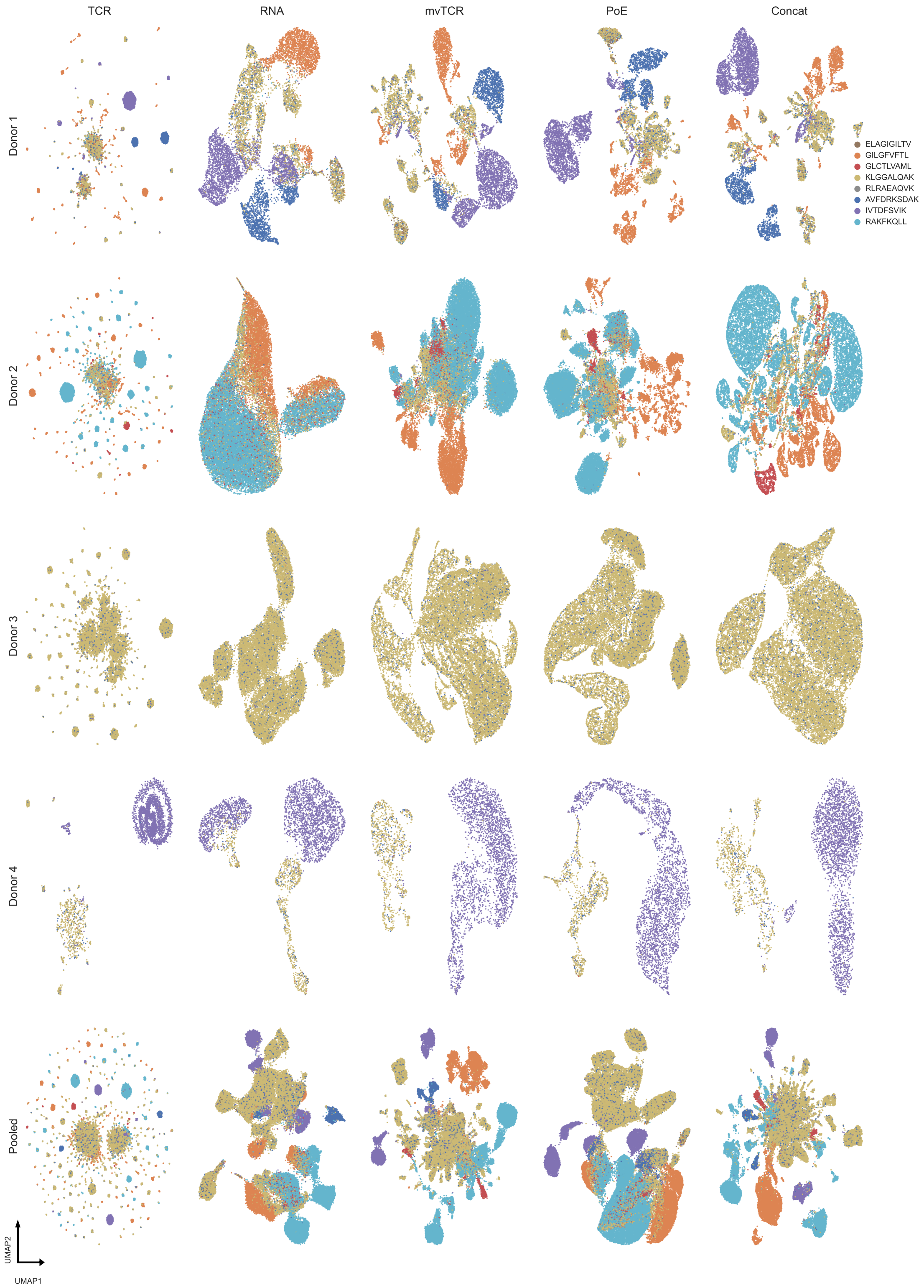

Supplementary Figure 2 | Embeddings of the 10x Genomics dataset. UMAP visualizations of the embeddings from different uni-modal (TCR and RNA) and multimodal models (mvTCR, PoE, Concatenation) colored by epitope specificity for donor 1-4 separately and all donors pooled.

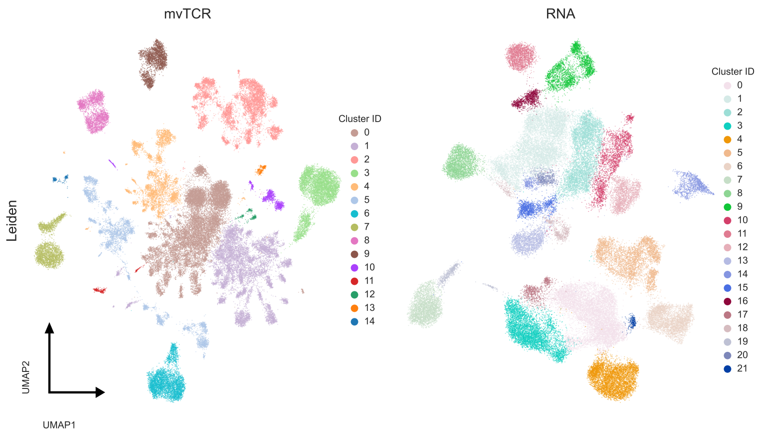

Supplementary Figure 3 | Leiden clusters in the 10x Genomics embeddings. Leiden clusters in the embeddings of the multimodal mvTCR and the unimodal RNA model for all donors pooled. The resolution was determined to maximize the Normalized Mutual Information (NMI) between the Leiden clusters and the pMHC-specificity.

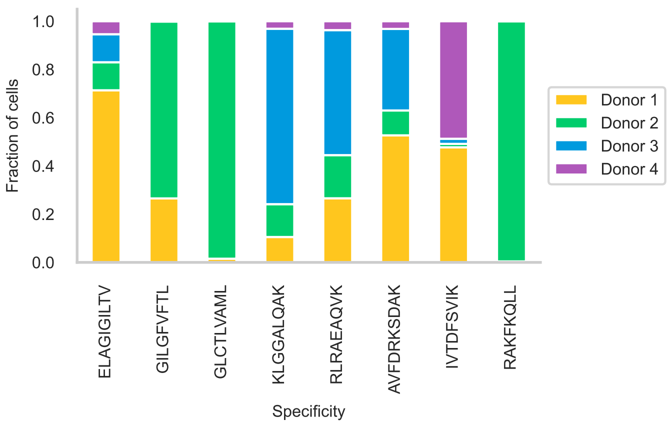

Supplementary Figure 4 | Specificity groups in the 10x dataset. Fraction of cells that belong to the different donors in the full 10x dataset separated by the different pMHC specificities.

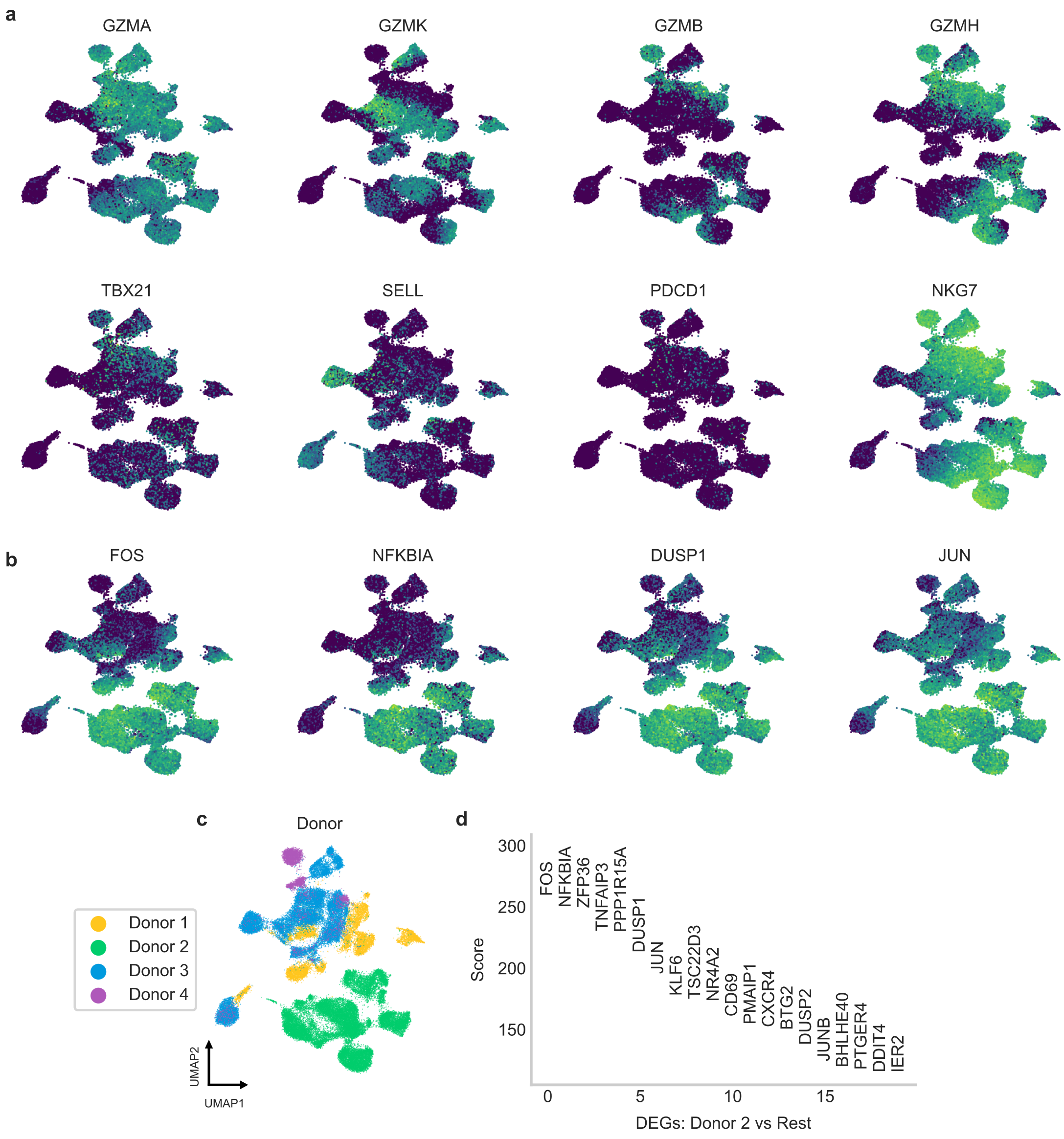

Supplementary Figure 5 | Donor effects in the 10x dataset. UMAP visualization of the RNA model colored by selected T cell marker genes (**a**), differentially expressed *ex vivo* activation signature genes (**b**), and donor ID (**c**). **d**, Differentially expressed genes of Donor 2 compared to the remaining dataset.

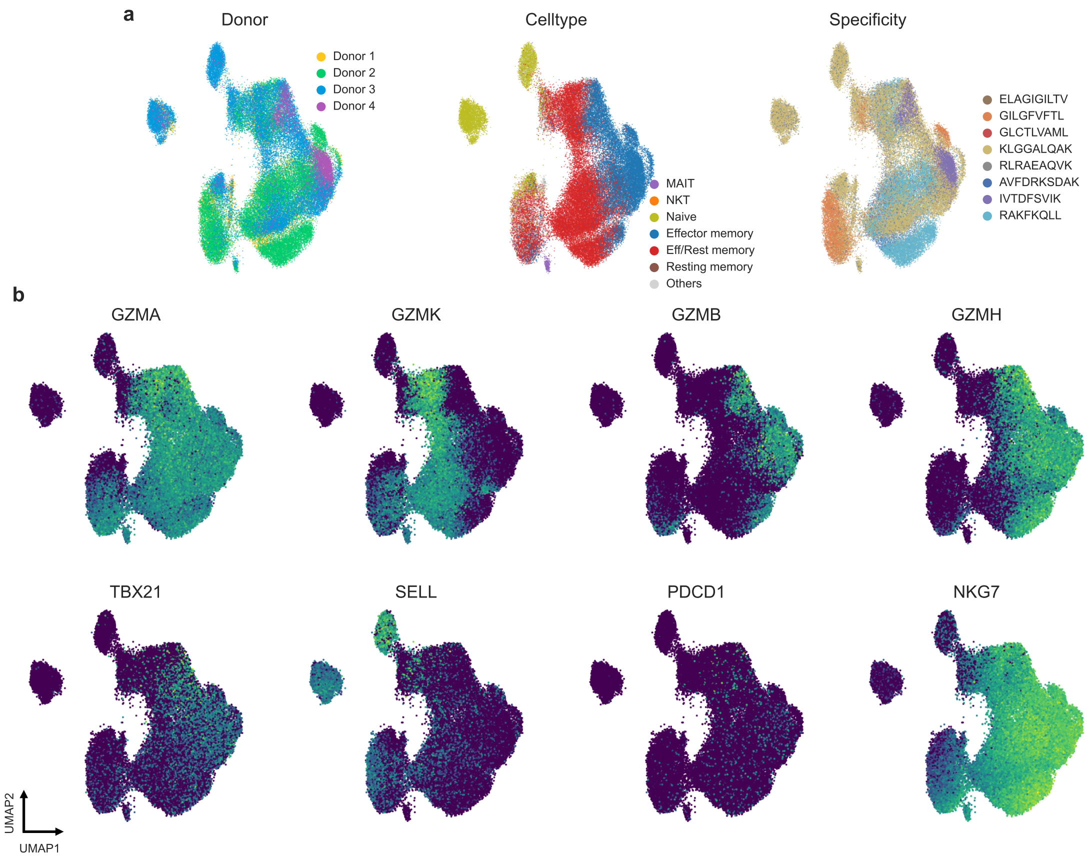

Supplementary Figure 6 | Harmony correction of the 10x dataset. UMAP visualization of the 10x dataset using Harmony [1] for batch correction colored by donor, cell type, and specificity (**a**) as well as selected T cell marker genes (**b**).

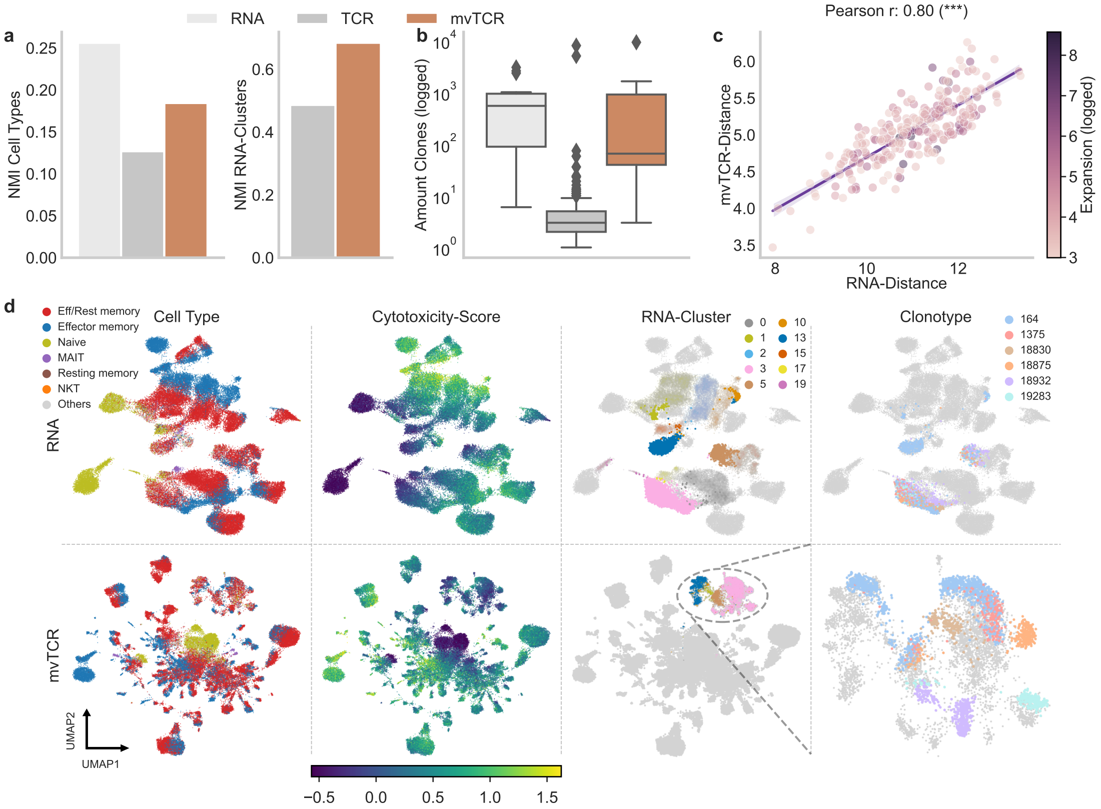

Supplementary Figure 7 | Transcriptomic information is conserved in the joint embedding. a, Clustering performance for cell type derived from the transcriptome and RNA clusters defined by the RNA model. **b**, Amount of clonotypes within each cluster ($n_{RNA} = 22$, $n_{TCR} = 214$, $n_{mvTCR} = 15$ clusters). The box plots indicates the data quartiles with the whiskers extending to the full distribution excluding outliers outside the 1.5 interquartile range while the median is indicated as a horizontal line. **c**, Correlation between the average distance within a clonotype of more than 20 cells in the RNA representation compared to the mvTCR model (p-values: $*<0.05$, $**<0.01$, $***<0.001$, $n = 247$ clonotypes). The line indicates the linear regression fit with the 95% confidence interval as error band. **d**, UMAP visualizations [2] comparing the embeddings of the transcriptomic and the mvTCR models colored by cell type, cytotoxicity-score [3], clusters defined in the RNA model that occur in mvTCR cluster 2, and the cells of the six largest clonotypes of this cluster. Cells in the RNA representation were highlighted fully for cells of the mvTCR cluster 2 and with reduced transparency for the remaining cells of the corresponding clusters. 18 outlier cells were removed from the zoomed UMAP of the mvTCR model colored by clonotype for visualisation purposes.

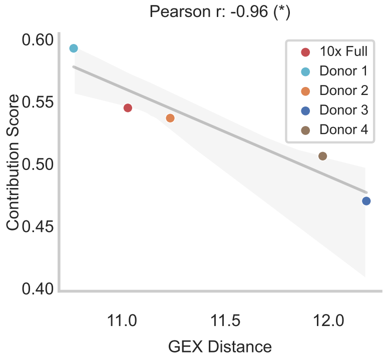

Supplementary Figure 8 | Dataset-specific modality contribution. Correlation in the 10x dataset between the TCR-Contribution averaged per donor and the average transcriptomic distance within large clonotypes consisting of 20 or more cells (p-value=0.0114, $n = 5$). The line indicates the linear regression fit with the 95% confidence interval as error band.

a

Model	F1-Score 10x Full	D1	D2	D3	D4	Minervina	Total	NMI 10x Full	D1	D2	D3	D4	Minervina	Total
**mvTCR**	0.80	0.82	0.84	0.89	0.78	0.79	**0.82**	0.50	0.58	0.41	0.00	0.52	0.44	**0.41**
**PoE**	0.80	0.75	0.86	0.89	0.78	0.78	**0.81**	0.43	0.54	0.36	0.01	0.51	0.42	**0.38**
**Concat**	0.82	0.72	0.81	0.89	0.78	0.79	**0.80**	0.46	0.55	0.39	0.00	0.71	0.42	**0.42**

b

Model	F1-Score 10x Full	D1	D2	D3	D4	Minervina	Total	NMI 10x Full	D1	D2	D3	D4	Minervina	Total
**mvTCR**	0.80	0.82	0.84	0.89	0.78	0.79	**0.82**	0.50	0.58	0.41	0.00	0.52	0.44	**0.41**
**Sup-Repr**	0.84	0.63	0.86	0.89	0.77	0.83	**0.80**	0.61	0.63	0.54	0.05	0.63	0.72	**0.53**
**Sup-Class**	0.51	0.43	0.62	0.50	0.28	0.78	**0.52**							

Supplementary Figure 9 | Additional specificity benchmarks. Capturing of pMHC specificity by atlas-query prediction (weighted F1-Score) and clustering (Normalized Mutual Information) on the 10x Genomics dataset for all donors (10x Full), donors 1-4 separately (D1-D4), and the Minervina dataset. Each score represents the average over $n = 5$ random splits. **a**, Comparison of the different mixture models. **b**, Comparison of the base model to the results directly obtained with the supervised classification head (Sup-Class) and its representation (Sup-Repr).

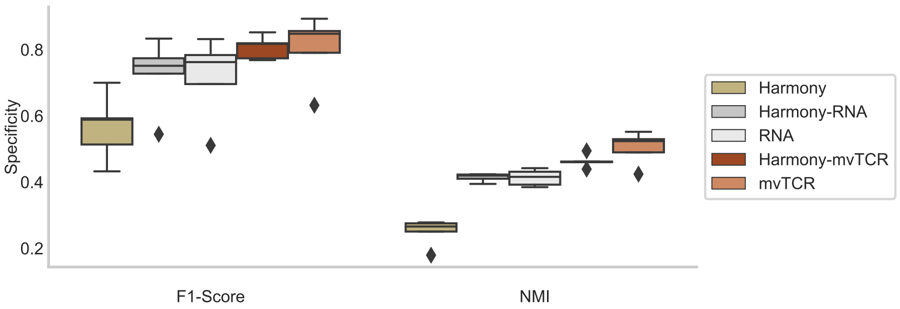

Supplementary Figure 10 | Benchmark using Harmony batch correction. Specificity prediction (F1-Score) and clustering performance (NMI) on the pooled 10x dataset on $n = 5$ splits using the Principal Components (PCs) provided by Harmony [1], a VAE model trained on these PCs (Harmony-RNA), a VAE model trained on these PCs and TCR sequences (Harmony-mvTCR), and the two original models introduced earlier (RNA, mvTCR). The box plots indicacte the data quartiles with the whiskers extending to the full distribution excluding outliers outside the 1.5 interquartile range while the median is indicated as a horizontal line.

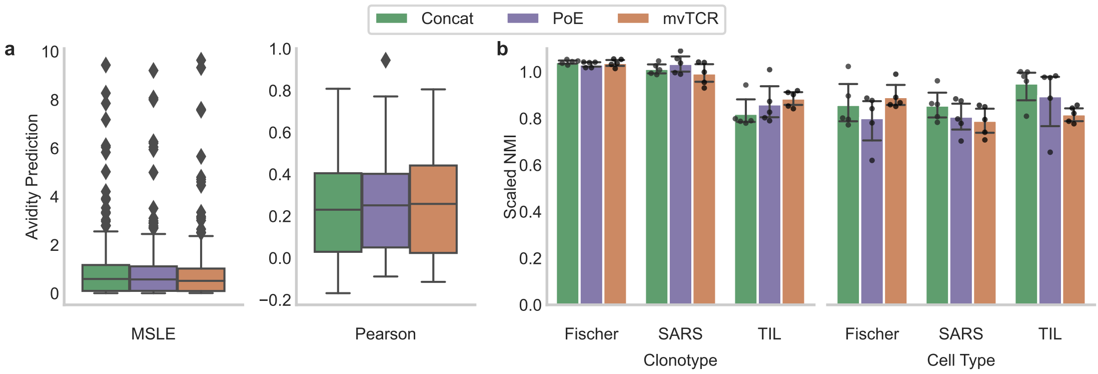

Supplementary Figure 11 | Additional tests on the different mixture models. **a**, Avidity prediction on the embedding of mvTCR, PoE, and Concatenation measured by mean squared logarithmic error (MSLE) and Pearson correlation for each of the five splits and eight specificity groups on the five versions of the 10x Genomics dataset ($n = 200$). The box plots indicacte the data quartiles with the whiskers extending to the full distribution excluding outliers outside the 1.5 interquartile range while the median is indicated as a horizontal line. **b**, Clustering performance (NMI, normalized by performance of underlying modality) of cell type defined on the transcriptome and clonotype defined on TCR sequence in the mvTCR embedding on the Fischer, SARS-CoV-2, and TIL datasets compared between the three mixture models for $n = 5$ splits. The bars represent the average metric score, while the error bars indicate the 95% confidence interval.

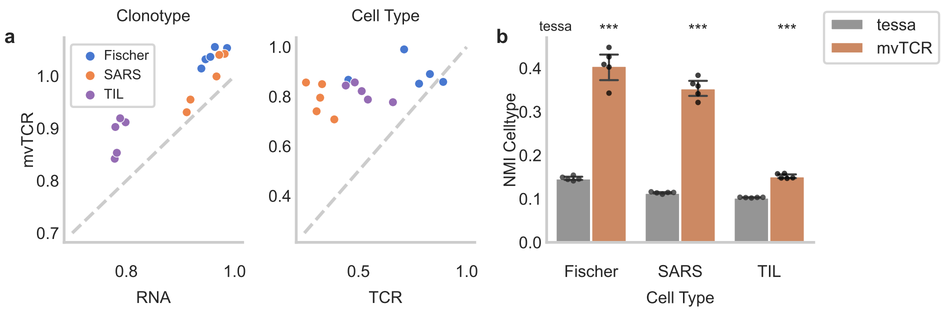

Supplementary Figure 12 | Conservation of modality-specific cell characteristics. **a**, Clustering performance (NMI, normalized by performance of underlying modality) of cell type defined on the transcriptome and clonotype defined on TCR sequence in the mvTCR embedding on the Fischer, SARS-CoV-2, and TIL datasets compared to the corresponding other modality. **b**, Comparison between mvTCR embedding trained only on the gene expression and the CDR3β sequence, and tessa [4] on the tasks defined in a. Statistical significance (p-values: p*<0.05, **<0.01, ***<0.001, baseline indicated left, $n = 5$ splits, $p_{Fischer} = 4.14 * 10^{-5}$, $p_{SARS} = 1.10 * 10^{-5}$, $p_{TIL} = 1.54 * 10^{-5}$) to the tessa algorithm is calculated via one-sided, paired t-test. The bars represent the average metric score, while the error bars indicate the 95% confidence interval.

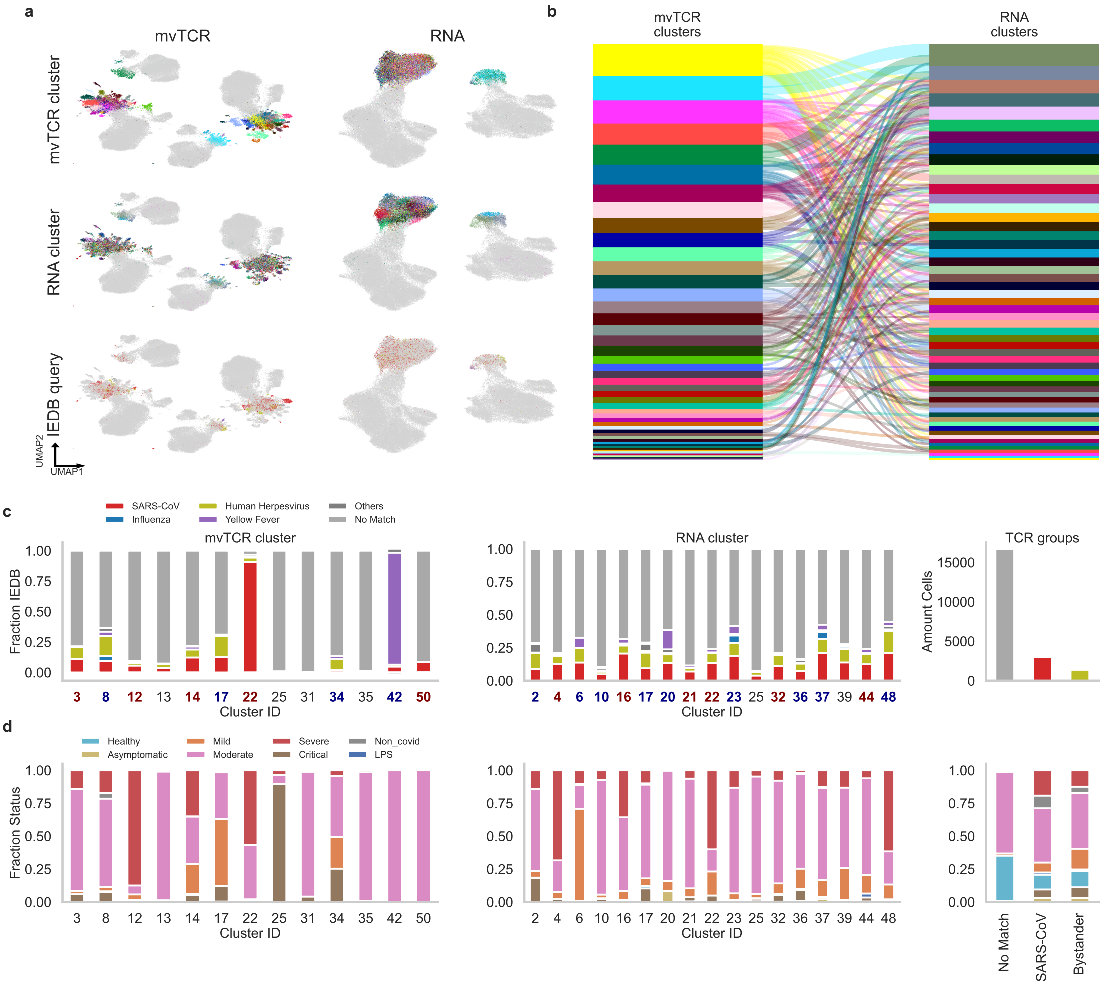

Supplementary Figure 13 | Comparison between mvTCR and unimodal clusters of the SARS-CoV-2 dataset. **a**, UMAP visualizations of the mvTCR and RNA embedding colored by clusters defined in the mvTCR representation, clusters defined on the RNA representation, and the annotated T cell specificity based on a sequence query to the IEDB. **b**, Distribution of the T cells from the mvTCR clusters in the RNA clusters. **c**, Proportion of cells assigned with specificity for the different clusters. For TCR queries, the total amount of cells is listed. **d**, Proportion of T cells from patients of the various severity degrees within the mvTCR clusters, RNA clusters, and TCR-specificity groups.

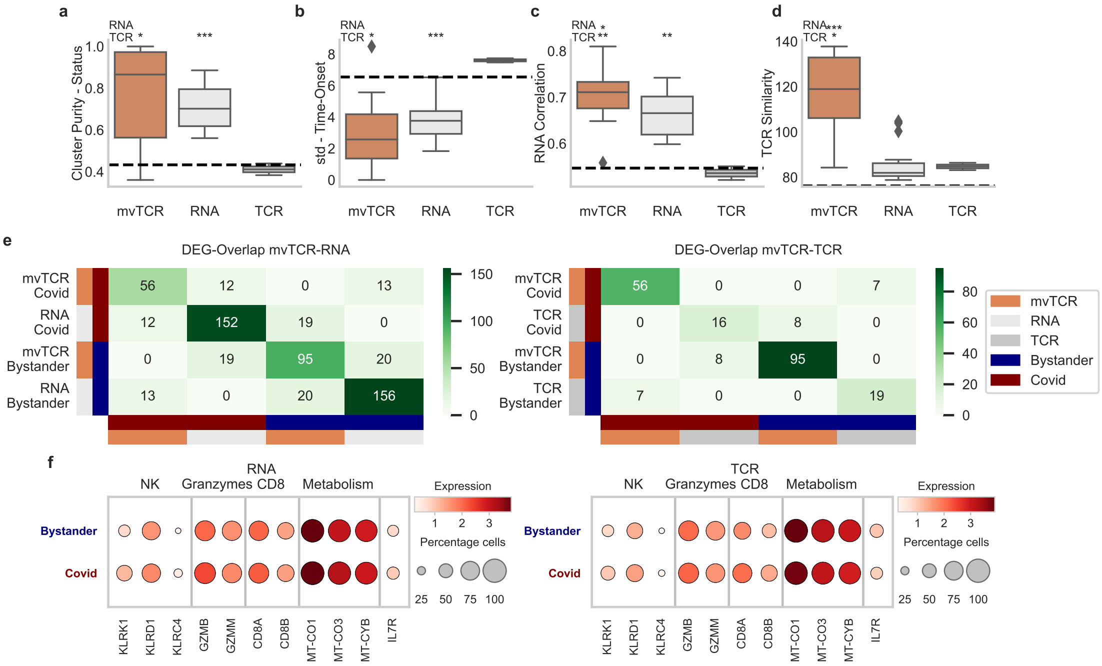

Supplementary Figure 14 | Quantitative comparison between unimodal and mvTCR analysis of the SARS-CoV-2 dataset., The cluster purity with respect to the cells patient status (**a**), standard deviation (std) of time after disease onset (**b**), pairwise gene expression correlation (**c**), and TCR similarity (**d**) between cells of the selected clusters in the mvTCR representation ($n_{mvTCR} = 13$), RNA representation ($n_{RNA} = 17$), and the TCR-specificity groups ($n_{TCR} = 2$). All box plots indicate the data quartiles while the whiskers extend to the extreme values excluding outliers outside the 1.5 interquartile range. The median is indicated as a horizontal line. The dashed horizontal line indicates the average of all CD8$^+$ effector T cells. Statistical significance (p-values: $*<0.05$, $**<0.01$, $***<0.001$, baseline indicated left, see Source Data) to the corresponding unimodal analysis is calculated via one-sided, paired t-test. **e**, Overlap of differentially expressed genes of SARS-CoV-2-specific and bystander T cells identified by mvTCR compared to the RNA embedding and TCR-specificity groups. **f**, Expression of selected genes during mvTCR analysis on the cells identified as SARS-Cov-2 reactive on the RNA or TCR information alone.

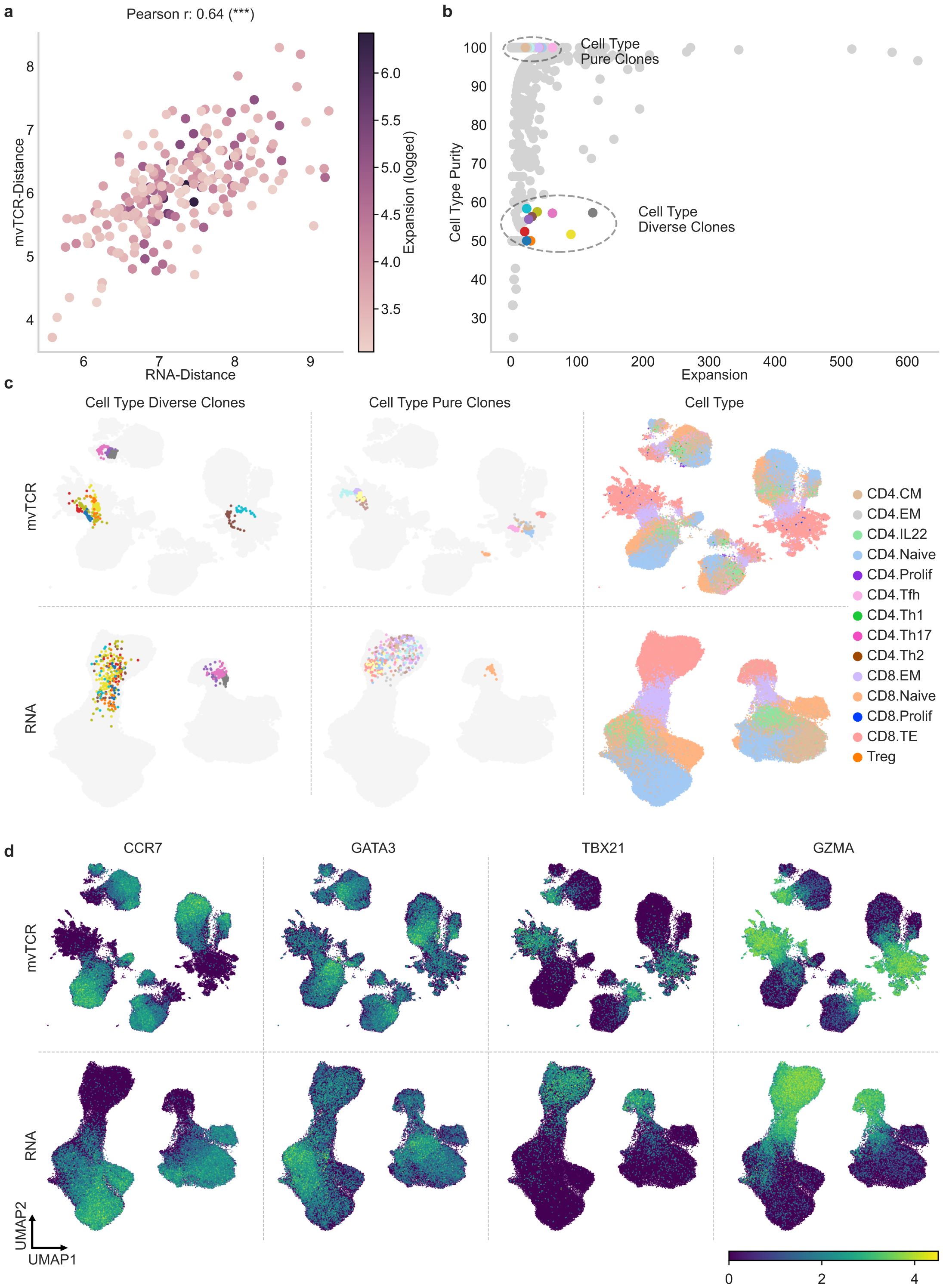

Supplementary Figure 15 | RNA variation is conserved by mvTCR. a, Correlation between the within-clonotype distance in the RNA-space (x-axis) and the mvTCR-space (y-axis). Each point is a clone with a minimum of 20 cells and is colored by the logarithm of the number of cells sharing that clone ($n = 222$ clonotypes, p-values: ***<0.001). **b**, Amount of each clone's cells and its cell-type purity, computed as the frequency of the most abundant cell type. The dashed line indicates clones with less than 20 cells and excluded from the analysis. The 10 clones with the highest and the lowest cell type purity are highlighted. **c**, UMAP representation of the mvTCR and the RNA colored by selected clonotypes and most frequent cell types. **d**, UMAP representation of the mvTCR and the RNA colored by naïve and CD4$^+$ and CD8$^+$ T cells activation markers.

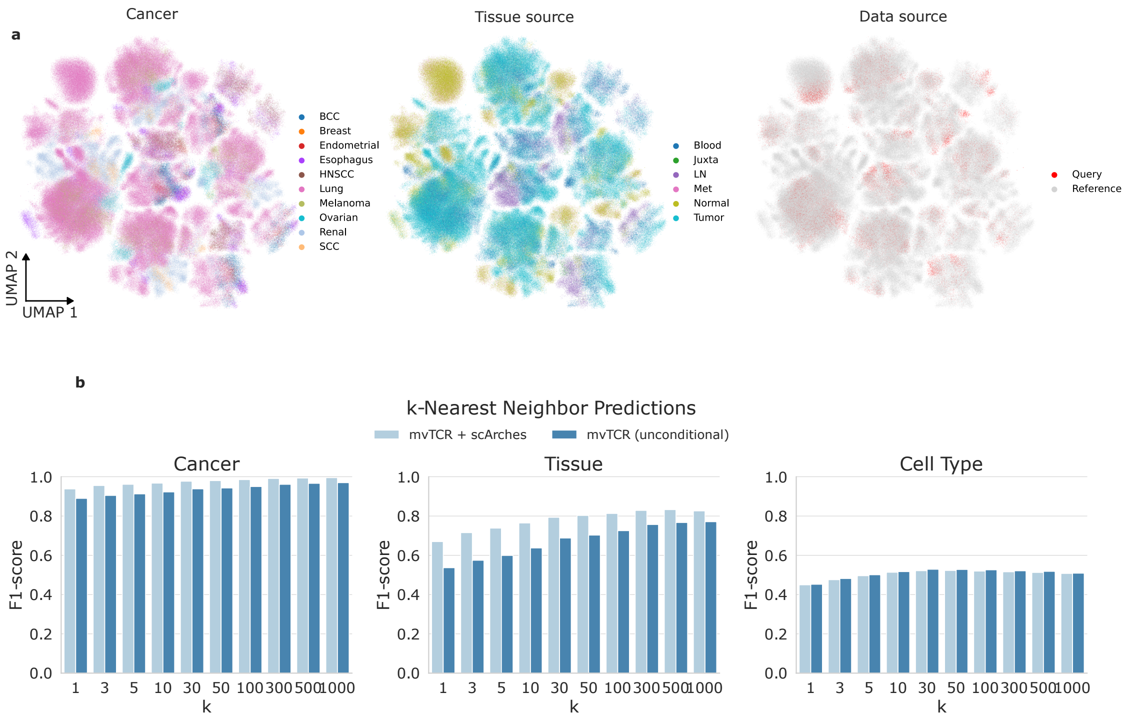

Supplementary Figure 16 | Embedding of the TIL dataset without scArches. a, UMAP visualizations of the joint embedding colored by cancer tissue, tissue type, and query vs. reference set. **b**, kNN predictions with varying k-values using the multimodal embedding as features to classify biological labels on the query dataset.

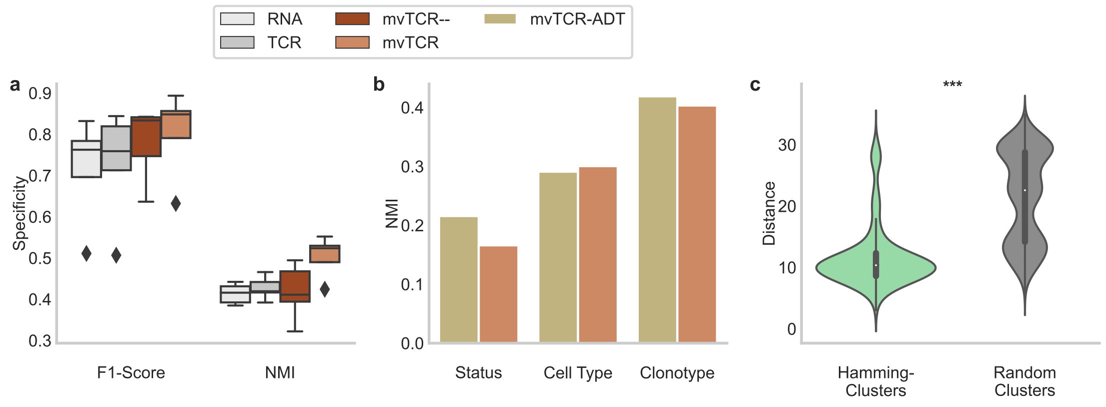

Supplementary Figure 17 | Extensions of mvTCR. a, Performance of mvTCR trained with simulated missing TCR-chains and GEX (mvTCR--) on the pooled 10x dataset for prediction and clustering compared to the base mvTCR model and the unimodal baselines ($n = 5$). **b**, Clustering of mvTCR with TCR, transcriptome, and surface protein markers (mvTCR-ADT) compared to the baseline model on the SARS-CoV-2 dataset. **c**, Average distances within clusters of expanded TCR clones with single amino-acid mutations in the CDR3β-chain compared to random clusters in the SARS-CoV-2 dataset (p-values: *<0.05, **<0.01, ***<0.001, $n = 2,992$). All box plots indicate the data quartiles with the whiskers extending to the full distribution excluding outliers outside the 1.5 interquartile range while the median is indicated as a white point.

References

[1] Korsunsky, I. *et al.* Fast, sensitive and accurate integration of single-cell data with harmony. *Nature methods* **16**, 1289–1296 (2019).

[2] McInnes, L., Healy, J. & Melville, J. Umap: Uniform manifold approximation and projection for dimension reduction. *arXiv preprint arXiv:1802.03426* (2018).

[3] Szabo, P. A. *et al.* Single-cell transcriptomics of human t cells reveals tissue and activation signatures in health and disease. *Nature communications* **10**, 1–16 (2019).

[4] Zhang, Z., Xiong, D., Wang, X., Liu, H. & Wang, T. Mapping the functional landscape of t cell receptor repertoires by single-t cell transcriptomics. *Nature methods* **18**, 92–99 (2021).
