# Supplementary material for: Multi-modal generative modeling for joint analysis of single-cell T cell receptor and gene expression data
Source: Nat Commun. 2024 Jul 3;15:5577. doi: 10.1038/s41467-024-49806-9 (PMC11220149; doi:10.1038/s41467-024-49806-9)
Supplement: Supplementary file 3 — Reporting Summary [file 41467_2024_49806_MOESM3_ESM.pdf]

nature portfolio

Corresponding author(s): Benjamin Schubert, Mohammad Lotfollahi

Last updated by author(s): 2024.05.06

Reporting Summary

Nature Portfolio wishes to improve the reproducibility of the work that we publish. This form provides structure for consistency and transparency in reporting. For further information on Nature Portfolio policies, see our Editorial Policies and the Editorial Policy Checklist.

Statistics

For all statistical analyses, confirm that the following items are present in the figure legend, table legend, main text, or Methods section.

n/a	Confirmed	
[ ]	[x]	The exact sample size (*n*) for each experimental group/condition, given as a discrete number and unit of measurement
[ ]	[x]	A statement on whether measurements were taken from distinct samples or whether the same sample was measured repeatedly
[ ]	[x]	The statistical test(s) used AND whether they are one- or two-sided<br>*Only common tests should be described solely by name; describe more complex techniques in the Methods section.*
[x]	[ ]	A description of all covariates tested
[ ]	[x]	A description of any assumptions or corrections, such as tests of normality and adjustment for multiple comparisons
[ ]	[x]	A full description of the statistical parameters including central tendency (e.g. means) or other basic estimates (e.g. regression coefficient) AND variation (e.g. standard deviation) or associated estimates of uncertainty (e.g. confidence intervals)
[ ]	[x]	For null hypothesis testing, the test statistic (e.g. *F*, *t*, *r*) with confidence intervals, effect sizes, degrees of freedom and *P* value noted<br>*Give P values as exact values whenever suitable.*
[x]	[ ]	For Bayesian analysis, information on the choice of priors and Markov chain Monte Carlo settings
[x]	[ ]	For hierarchical and complex designs, identification of the appropriate level for tests and full reporting of outcomes
[ ]	[x]	Estimates of effect sizes (e.g. Cohen's *d*, Pearson's *r*), indicating how they were calculated

Our web collection on statistics for biologists contains articles on many of the points above.

Software and code

Policy information about availability of computer code

Data collection	Publicly available datasets were downloaded from their respective repositories (see Data statement).
Data analysis	The software code including tutorials is available at https://github.com/SchubertLab/mvTCR. The code to reproduce the results of this manuscript can be accessed under https://github.com/SchubertLab/mvTCR_reproducibility. All trained models used for this manuscript can be downloaded from Zenodo via https://doi.org/10.5281/zenodo.7215447.<br>The method and analysis was implemented in python 3.8.8 using pytorch = 1.8.0, scanpy = 1.7.0, anndata = 0.7.6, scirpy = 0.11, pandas = 1.2.3, numpy = 1.20.3, scikit-learn = 0.24.1, scrublet==0.2.3, optuna = 2.10.0, umap-learn==0.5.1, and leidenalg = 0.8.4.

For manuscripts utilizing custom algorithms or software that are central to the research but not yet described in published literature, software must be made available to editors and reviewers. We strongly encourage code deposition in a community repository (e.g. GitHub). See the Nature Portfolio guidelines for submitting code & software for further information.

Data

Policy information about availability of data

All manuscripts must include a data availability statement. This statement should provide the following information, where applicable:

- Accession codes, unique identifiers, or web links for publicly available datasets
- A description of any restrictions on data availability
- For clinical datasets or third party data, please ensure that the statement adheres to our policy

All datasets used in this paper are publicly available. The 10x dataset was accessed from the 10x website under the Section Application Note - A New Way of Exploring Immunity [https://www.10xgenomics.com/datasets] (accessed March, 7th, 2021). The Minervina dataset was accessed from Zenodo under the accession code 6231854 [https://doi.org/10.5281/zenodo.6232103]. The the SARS-CoV-2 dataset was accessed from the Covid-19 Cell Atlas under the section Section COVID-19 PBMC Ncl-Cambridge-UCL [https://www.covid19cellatlas.org/index.patient.html] (accessed February, 2nd, 2022). The Fischer dataset was accessed from the NCBI GEO under the accession number GSE171037 [https://www.ncbi.nlm.nih.gov/geo/query/acc.cgi?acc=GSE171037]. The samples contained in TIL dataset stem from a collection of studies. A processed version of this data was downloaded as described in https://github.com/ncborcherding/utility (accessed December, 20th, 2021). Source data are provided with this paper.

Research involving human participants, their data, or biological material

Policy information about studies with human participants or human data. See also policy information about sex, gender (identity/presentation), and sexual orientation and race, ethnicity and racism.

Reporting on sex and gender	Does not apply for the manuscript as we worked with published data and did not conduct any studies ourselves.
Reporting on race, ethnicity, or other socially relevant groupings	N.A.
Population characteristics	N.A.
Recruitment	N.A.
Ethics oversight	N.A.

Note that full information on the approval of the study protocol must also be provided in the manuscript.

Field-specific reporting

Please select the one below that is the best fit for your research. If you are not sure, read the appropriate sections before making your selection.

☒ Life sciences ☐ Behavioural & social sciences ☐ Ecological, evolutionary & environmental sciences

For a reference copy of the document with all sections, see nature.com/documents/nr-reporting-summary-flat.pdf

Life sciences study design

All studies must disclose on these points even when the disclosure is negative.

Sample size	We used publicly available datasets. To this end, we collected a non-exhaustive list of single-cell experiments of paired scRNA and scTCR, for which we used the complete dataset.
Data exclusions	From published dataset, we used the full dataset.
Replication	The benchmark experiments on the 10x, Minervina, Fischer, SARS-CoV-2, and TIL dataset (only for clustering on celltype and clonotype) were conducted on n=5 different dataset splits. Source code (https://github.com/SchubertLab/mvTCR_reproducibility) is provided for all experiments for reproducability.
Randomization	Does not apply for the manuscript as we worked with published data and did not conduct any studies ourselves.
Blinding	Does not apply for the manuscript as we worked with published data and did not conduct any studies ourselves.

Reporting for specific materials, systems and methods

We require information from authors about some types of materials, experimental systems and methods used in many studies. Here, indicate whether each material, system or method listed is relevant to your study. If you are not sure if a list item applies to your research, read the appropriate section before selecting a response.

Materials & experimental systems

n/a	Involved in the study
☒	☐ Antibodies
☒	☐ Eukaryotic cell lines
☒	☐ Palaeontology and archaeology
☒	☐ Animals and other organisms
☒	☐ Clinical data
☒	☐ Dual use research of concern
☒	☐ Plants

Methods

n/a	Involved in the study
☒	☐ ChIP-seq
☒	☐ Flow cytometry
☒	☐ MRI-based neuroimaging

Plants

Seed stocks	No plant material was used in this study.
Novel plant genotypes	N.A.
Authentication	N.A.
