# Supplementary material for: Multi-modal generative modeling for joint analysis of single-cell T cell receptor and gene expression data
Source: Nat Commun. 2024 Jul 3;15:5577. doi: 10.1038/s41467-024-49806-9 (PMC11220149; doi:10.1038/s41467-024-49806-9)
Supplement: Supplementary file 4 — Description of Additional Supplementary Files [file 41467_2024_49806_MOESM4_ESM.pdf]

Description of Additional Supplementary Files

File Name: Supplementary Data 1

Description: Cluster selection on the SARS-CoV-2 dataset. Statistical analysis of the IFN response score across clusters, similarity of transcriptome and TCRs of CD8+ T effector cells within selected clusters, and comparison of the mvTCR and unimodal clusters.

File Name: Supplementary Data 2

Description: HLA annotation for the SARS-CoV-2 dataset. Information about HLA type for each donor in the SARS-CoV-2 dataset.

File Name: Supplementary Data 3

Description: IEDB-based Epitope Query for selected clusters of the SARS-CoV-2 dataset. Pairs of TCRs and epitopes from selected clusters of the SARS-CoV-2 dataset derived from a database query to the IEDB.

File Name: Supplementary Data 4

Description: DEG analysis of CD8+ T effector cells in the SARS-CoV-2 dataset. Log-fold change, p-values, and adjusted p-values of the genes comparing disease-specific and bystander clusters in the SARS-CoV-2 dataset for TCR groups, mvTCR, and gene expression clusters.
